# The ideological divide and climate change opinion: “top-down” and “bottom-up” approaches

**DOI:** 10.3389/fpsyg.2014.01458

**Published:** 2014-12-18

**Authors:** Jennifer Jacquet, Monica Dietrich, John T. Jost

**Affiliations:** ^1^Department of Environmental Studies, New York University, New York, NY, USA; ^2^Department of Psychology, New York University, New York, NY, USA

**Keywords:** U.S. political psychology, ideology, climate change, system justification, liberal-conservative divide

## Abstract

The United States wields disproportionate global influence in terms of carbon dioxide emissions and international climate policy. This makes it an especially important context in which to examine the interplay among social, psychological, and political factors in shaping attitudes and behaviors related to climate change. In this article, we review the emerging literature addressing the liberal-conservative divide in the U.S. with respect to thought, communication, and action concerning climate change. Because of its theoretical and practical significance, we focus on the motivational basis for skepticism and inaction on the part of some, including “top-down” institutional forces, such as corporate strategy, and “bottom-up” psychological factors, such as ego, group, and system justification. Although more research is needed to elucidate fully the social, cognitive, and motivational bases of environmental attitudes and behavior, a great deal has been learned in just a few years by focusing on specific ideological factors in addition to general psychological principles.

## INTRODUCTION

The scientific community exhibits widespread agreement about anthropogenic climate change and the need to reduce greenhouse gas emissions (Anderegg et al., 2010). For a number of reasons, including the intergenerational nature of climate change policy, whereby sacrifices made today will not yield dividends for decades to come (Schelling, 1995; Bazerman, 2006; Jacquet et al., 2013), greenhouse gas reductions will not be accomplished easily. Despite the fact that obstacles are universal, there is considerable variation in the degree to which individual and corporate actors (including nation-states) have sought to mitigate fossil fuel use—a fact that should, and does, interest social scientists.

Due to its disproportionate global influence in terms of carbon dioxide emissions (second only to China) as well as its role in affecting international climate policy, the U.S. stands out as an especially important context in which to examine social, psychological, and political dynamics. In 2009, a task force of the American Psychological Association (APA) identified numerous reasons for the public’s lack of urgency on the issue, including old habits, feelings of personal insignificance, uncertainty about the severity of climate changes, mistrust of information, the belief that the costs of climate change will occur later in the future than scientists expect, and high rates of denial and skepticism (Swim et al., 2010). Indeed, skepticism about climate change is higher in the U.S. than in other countries (Anderegg et al., 2010; Poortinga et al., 2011; Engels et al., 2013)—and this fact itself requires deeper explanation. It seems especially pertinent that denial and skepticism are not uniformly distributed across the political landscape; conservatives express greater skepticism about climate change and more opposition to climate-related policies than liberals (e.g., Weber and Stern, 2011; Liu et al., 2014).

In the APA report, Swim et al. (2010) cited just two studies addressing the ideological divide over climate change policy (Dunlap and McCright, 2008; Hardisty et al., 2010). Since that time, a literature has emerged to analyze liberal-conservative differences in climate-related attitudes and behaviors, including studies that have highlighted motivational factors that help to explain the ideological divide and its implications for political action (or inaction) when it comes to climate change. Jost et al. (2009) proposed that ideological outcomes are typically the joint product of “top-down” elite-driven forms of communication (i.e., the discursive superstructure) and “bottom-up” psychological factors that make citizens more or less receptive to those forms of communication (i.e., the motivational substructure). In an effort to integrate “top-down” and “bottom-up” approaches, we review recent research on the U.S. ideological divide that is focused specifically on climate change (rather than environmental concerns more broadly, but see, e.g., Dunlap et al., 2001; Xiao and McCright, 2007; Feygina et al., 2010; Liu et al., 2014).

## THE IDEOLOGICAL DIVIDE: PUBLIC OPINION DATA

Since the 1980s, U.S. political leaders have been resistant—symbolically and operationally—to domestic action and international cooperation on climate change (Jamieson, 2014). Polarization among the American public has been on the rise since the 1990s (Guber, 2013). In a 2010 Gallup survey of 1,014 adults in the U.S., 74% of liberals agreed that “effects of global warming are already occurring,” whereas only 30% of conservatives concurred (Jones, 2010). Public opinion surveys of 1,024 Americans in 2012 revealed that 42% contend that climate change claims are “generally exaggerated” and that political conservatives are more skeptical of climate change than liberals (Saad, 2012). Even among Republicans, there appears to be an ideological split: a survey of 1,504 Americans in October 2013 found that 61% of non-Tea Party Republicans believe that there is solid evidence of global warming, as compared to only 25% of Tea Party Republicans (Pew Research Center, 2013).

While some surveys suggest broad support for certain climate change-related policies (e.g., tax breaks for renewable energies; Krosnick and MacInnis, 2013), other studies reveal pervasive ideological cleavages. In a survey of 209 Pittsburgh residents concerning fossil fuel consumption, Republicans were 4.5 times and Independents were 4.2 times more likely than Democrats to reject regulations proposed to limit SUVs and trucks (Attari et al., 2009). Gromet et al. (2013) surveyed 657 U.S. residents and found that people who identified themselves as politically conservative were less supportive of investment in energy-efficient technology than those who were more liberal. Based on a survey of 375 residents from Michigan, Bidwell (2013) concluded that opposition to commercial wind farms was “fueled by conservatism.”

Natural field experiments also highlight the extent of ideological division. Providing households—which account for approximately 38% of U.S. total emissions (Dietz et al., 2009)—weekly or monthly feedback about their home energy use (compared to that of their neighbors) can lower overall energy consumption (Schultz et al., 2007). Costa and Kahn (2013) analyzed data from 81,722 homes (48,058 of which were in a control group) over the course of nearly 3 years and connected homeowners with voter registration records (i.e., party affiliation). Compared to Democrats, Republicans were more likely to opt out of the energy program, less likely to indicate that they liked the home energy reports and found them useful, and were less likely to reduce their energy consumption during the course of the intervention.

## “TOP-DOWN” FACTORS: INSTITUTIONAL EFFECTS ON COMMUNICATION AND DISCOURSE

**FIGURE 1:**
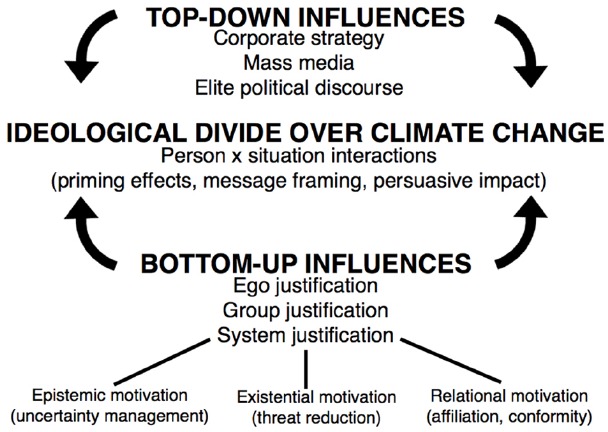
**Contributions of top-down and bottom-up influences to the ideological divide over climate change**.

Evidence suggests that there are clear “top-down” institutional forces at work when it comes to skepticism about climate change and political acquiescence, and that these forces exacerbate the ideological divide (see Figure 1). Sociologists Dunlap and McCright (2011) link the rise of climate change denial to corporate and right-wing strategists, such as Richard Mellon Scaife and the Koch brothers (who have given at least $48 million—half of that since 2005—to groups that actively deny global warming). Scholars and investigative journalists have become increasingly concerned about the historical role of corporations and politicians in deceiving the public about the risks of a wide range of behaviors associated with tobacco use, pollution, and climate change (e.g., Michaels, 2008; Oreskes and Conway, 2010).

Evidence from cross-national studies confirms that information communicated in the U.S. is distinct from what is communicated in the rest of the world. Bailey et al. (2014) compared climate change coverage in 2001 and 2007 in U.S. (*New York Times* and *Wall Street Journal*) and Spanish newspapers (*El Mundo* and *El Pais*) and found that U.S. newspapers used twice as much “hedging” language—words that suggest uncertainty (e.g., “inaccurate” or “speculative”). An analysis of 2,064 print media articles spanning six countries (Brazil, China, France, India, U.K., and the U.S.) from November 2009 to February 2010 revealed that the U.S. had the highest proportion of articles—one-third—expressing skeptical positions about climate change (Painter and Ashe, 2012).

Within the U.S., print media between 1998 and 2002 expressed more uncertainty about climate change than scientists registered (Boykoff and Boykoff, 2004). Content analysis of media in subsequent years has underscored high variability among news outlets. Studies comparing cable news television channels (i.e., Fox News, CNN, and MSNBC) demonstrated that Fox News has emphasized scientific uncertainty more than other networks and has focused more on stories that question the existence of human-caused climate change (Feldman et al., 2012). Elsasser and Dunlap (2013) analyzed 203 opinion editorials written by 80 U.S. conservative columnists published between 2007 and 2010 and found that all of them expressed doubts about climate change and/or climate science. Hmielowski et al. (2014) performed longitudinal research, surveying 2,497 U.S. residents in the fall of 2008 and 1,036 in a follow-up survey in the spring of 2011. The researchers discovered that the more individuals reported using conservative media, the less certain they were that climate change was real. Moreover, conservative media use was negatively associated with trust in science over time, suggesting one powerful way in which mass media influences beliefs.

## IDEOLOGICAL DIFFERENCES IN PROCESSING “TOP-DOWN” INFORMATION

Several studies have investigated the ways in which “top-down” forms of elite communication (and framing) interact with “bottom-up” factors such as the ideological inclinations of the audience. This work suggests that exposure to the same information can produce divergent effects—as a function of the message recipient’s political orientation—when it comes to attitudes about climate change. For example, an ideological divide was readily apparent in response to the 2007 report of the Intergovernmental Panel on Climate Change (IPCC). Budescu et al. (2012) asked 556 Americans to interpret the report’s use of words (rather than numerical percentages) to describe risk probabilities. Overall, respondents underestimated the problem of climate change as characterized in the report. For instance, the phrase “very likely,” which was intended to convey a probability of greater than 90% in statements such as “it is very likely that hot extremes, heat waves, and heavy precipitation events will continue to become more frequent,” was interpreted, on average, as suggesting a 62% likelihood. The underestimation effect was especially dramatic among political conservatives, who interpreted “very likely” as reflecting a probability of approximately 50%. A web experiment involving 400 Americans revealed that for conservatives the phrase “global warming” was associated with certain outcomes (such as rising temperatures and melting ice), whereas the phrase “climate change” was not; for liberals, there were no such differences in association (Schuldt and Roh, 2014).

Hardisty et al. (2010) studied 337 Americans to determine the effects of framing an environmental cost as a “tax” or an “offset” when it came to the (hypothetical) purchase of an airline ticket that included a surcharge for carbon dioxide emissions. Participants evaluated a regular ticket and a more expensive ticket similarly when the costlier ticket was framed as including an “offset,” but Republicans and Independents were significantly less approving of the costlier ticket when it included a “tax.” Only 23% of Republicans selected the more expensive option with the environmental “tax,” as compared to 56% of Republicans who selected the same ticket when it contained an “offset.” (Most Democrats were supportive of the surcharge regardless of whether it was described as an “offset” or “tax”).

Gromet et al. (2013) provided participants with $2 and asked them to purchase (and take home) one of two light bulbs—either an incandescent bulb or a compact fluorescent light (CFL) bulb, which is considered a more environmentally friendly choice. All participants were given information about the advantages of purchasing CFLs over incandescent bulbs, such as energy and cost savings as well as a longer lifespan. When experimenters made both the CFL and incandescent bulbs the same price ($0.50), nearly all participants (of all political stripes) purchased the CFL, regardless of whether it was explicitly labeled as “good for the environment” or left unlabeled. When the CFL was priced at three times that of the incandescent bulb (which reflects current pricing in the U.S.), conservatives and moderates were less likely to purchase the CFL when it was labeled as “good for the environment” than when it was not. Liberals showed no such difference. These findings suggest that more conservative individuals may forgo future cost savings to avoid projecting the image of an environmentally concerned citizen. Sociologists doing ethnographic work have similarly concluded that describing renewable technologies such as solar energy as “green” appears to limit the adoption of these products among political conservatives (Schelly, 2014).

## “BOTTOM-UP” FACTORS: EGO, GROUP, AND SYSTEM JUSTIFICATION MOTIVATION

Why would exposure to the same information elicit divergent responses from liberals and conservatives? Recent work at the intersection of sociology, psychology, and political science has emphasized the role of “motivated reasoning” (e.g., Taber and Lodge, 2006). It may be useful to distinguish among three motives that can shape the processing of scientific (and other) information, namely (a) ego (or self) justification, (b) group justification, and (c) system justification (Jost et al., 2013).

For over 30 years, researchers have understood that individuals engage in “biased assimilation,” so that they readily absorb new information that upholds the validity of their pre-existing beliefs and opinions while resisting new information that might challenge them (e.g., Lord et al., 1979; Ditto and Lopez, 1992). With respect to controversial political issues, Taber and Lodge (2006) demonstrated that citizens often exhibit “motivated skepticism”—using double standards to judge attitudinally incongruent arguments as weaker than attitudinally congruent arguments. This phenomenon might help to explain why respondents to Gallup surveys in 1990, 2000, and 2010 who felt that they understood the issue of climate change well were found to be more rather than less polarized in terms of environmental concern (Guber, 2013). Such ego-defensive tendencies, which are consistent with Festinger’s (1957) cognitive dissonance theory, serve the goal of preserving the individual’s self-esteem, insofar as it is easier to persist in the assumption that one’s opinions are correct.

In many cases, it may be difficult to disentangle ego and group justification motives for processing information in a selective or distortive manner. This is because many cherished beliefs are linked to membership in a social group (Tajfel and Turner, 1986) or political party (Cohen, 2003) or cultural background (Kahan et al., 2012; Kahan, 2013). Thus, an experiment conducted by Hart and Nisbet (2012) demonstrated that exposure to scientific information increased support for climate mitigation policies among Democrats, whereas exposure to the same information decreased support among Republicans. Another experiment (conducted in Australia) revealed that increasing the cognitive salience of political identification caused “right-wing” individuals to express more skepticism about climate change (Unsworth and Fielding, 2014). Kahan et al. (2012) investigated the climate change attitudes of 1,540 U.S. citizens and observed that greater levels of scientific and mathematical competence predicted increased polarization, suggesting that individuals may have been using their cognitive resources to bolster their own pre-existing opinions or those of their political party rather than engaging in a process of learning and updating on the basis of exposure to new information.

According to system justification theory, people are not only motivated to defend and bolster the interests and esteem of their personal self-concept and the social groups to which they belong; they are also motivated to defend and bolster aspects of the social, economic, and political systems on which they depend (Jost et al., 2004). This motivation, which is more explicitly ideological than ego or group justification motivation, tends to favor conservative ways of thinking and behaving, insofar as it activates the goal to justify the status quo. At the same time, there are important situational and dispositional sources of variability in the strength of system justification motivation. Some individuals, for instance, are chronically higher than others in psychological needs to reduce uncertainty and threat, and they are generally more driven to maintain pre-existing institutions, traditions, and social arrangements (e.g., Jost et al., 2009; Hennes et al., 2012).

Studies show that conservatives are indeed more strongly motivated by system justification concerns (e.g., Jost et al., 2008; Vainio et al., 2014) and that ideological differences in economic system justification help to explain why conservatives are more skeptical about climate change and less supportive of environmental action, in comparison with liberals and moderates (Feygina et al., 2010; Campbell and Kay, 2014; Leviston and Walker, 2014). Consistent with these results, Lewandowsky et al. (2013) surveyed 1,377 visitors to climate blogs and observed that rejection of climate science was predicted by endorsement of free market ideology.

Likewise, nationally representative surveys conducted in Australia demonstrated that system justification in the economic domain was negatively associated with support for carbon pricing and other pro-environmental initiatives. Economic system justification was also associated with decreased moral engagement concerning environmental issues and—consistent with the “palliative function” of system justification—decreased negative affect concerning climate change (Leviston and Walker, 2014).

A study of university students in Finland revealed that perceptions of climate change as a threat to the national system and right-wing orientation predicted system justification in general as well as justification of the food distribution system in Finland. System justification, in turn, was associated with denial of anthropogenic climate change, decreased knowledge about climate-friendly food choices, and a decreased willingness to make climate-friendly food choices (Vainio et al., 2014).

Hennes et al. (2014) demonstrated that when system justification motivation was temporarily activated, participants exhibited biased memory for scientific information and greater skepticism about climate change. More specifically, when participants were made to feel especially dependent on the social and economic system, they were prone to underestimate the proportion of carbon emissions that were caused by human activity (as reported in a newspaper article they had read earlier in the session). It is worth emphasizing that the memory biases elicited by system justification motivation tended to minimize problems associated with climate change and exonerate the overarching socioeconomic system. Thus, an additional (and often underappreciated) factor contributing to motivated reasoning about climate change is system justification motivation.

## CONCLUDING REMARKS

We have reviewed recent work in sociology, psychology, and political science that illuminates both “top-down” and “bottom-up” factors contributing to the ideological divide concerning climate change (see Figure 1). Although systematic research on this topic is only a few years old, there have been important advances. Institutional approaches emphasize the importance of “top-down” forms of elite communication, such as those related to corporate strategy, conservative think tanks, and mainstream media. Behavioral approaches focus on “bottom-up” processes, such as ego, group, and system justification motives, all of which are capable of contributing to polarization over climate change. We wish to point out that “top-down” and “bottom-up” factors are compatible and very often mutually reinforcing (see also Jost et al., 2009).

At the same time, it is clear that certain ways of framing messages are more effective than others when it comes to encouraging support for climate change policies (e.g., Feygina et al., 2010; Hardisty et al., 2010; Feinberg and Willer, 2011; Bain et al., 2012; Campbell and Kay, 2014). Johnson (2012) has argued that climate change communication is often ineffective because there is too much “fear messaging” and not enough “self-efficacy messaging,” which encourages people to feel that they possess significant control over the situation. Fear messaging seems to increase recipients’ needs for cognitive closure in general as well as their affinity for conservative labels and policies (Thórisdóttir and Jost, 2011), and conservatives tend to be more sensitive to threatening messages in the first place (Jost et al., 2003; Hibbing et al., 2014). Therefore, a little fear may go a long way, and it may induce citizens to respond defensively and engage in denial and minimization rather than facing up to environmental problems (Feygina et al., 2010; Jost and Hennes, 2013).

Nevertheless, focusing exclusively on message framing is likely to address proximate rather than ultimate causes of the ideological divide, which presumably include top-down, discursive structures as well as bottom-up, psychological functions. Few studies to date have isolated precise causal mechanisms linking political ideology to environmental attitudes and behaviors (but see Hennes et al., 2014, for an experimental attempt). We hope and anticipate that the demonstration of cause-effect relationships will become a higher priority in future research on the psychology of climate change. In the meantime, policy makers and concerned citizens will need to be more attentive to and effective in managing ideological processes and outcomes if the United States and other leading nations are to move beyond the present stalemate over climate change policy.

### Conflict of Interest Statement

The authors declare that the research was conducted in the absence of any commercial or financial relationships that could be construed as a potential conflict of interest.
